# Effect of dietary Anabaena supplementation on nutrient utilization, metabolism and oxidative stress response in *Catla catla* fingerlings

**DOI:** 10.1038/s41598-024-78234-4

**Published:** 2024-11-09

**Authors:** S. R. Mule, Dilip Kumar Singh, Patekar Prakash, Swapnil Ananda Narsale, M. D. Aklakur, Parimal Sardar, Gouranga Biswas, Sujata Sahoo, Manish Jayant, Samikshya Mishra

**Affiliations:** 1 ICAR - Central Institute of Fisheries Education, Mumbai, 400061 India; 2 ICAR- Central Institute of Fisheries Education, Kolkata Centre, Kolkata, West Bengal 700091 India

**Keywords:** Anabaena, Blue-green algae (BGA), *Catla catla*, Digestibility, Growth performance, Physio-metabolic responses, Physiology, Zoology, Environmental sciences

## Abstract

A 60-day feeding trial was conducted to evaluate the effects of dietary Anabaena blue-green algae (ABGA) meal on the growth performance, digestibility, and physio-metabolic responses of *Catla catla* fingerlings (initial average weight 9.45 ± 0.15 g). Six iso-nitrogenous (30% crude protein) and iso-caloric (378.09 Kcal. digestible energy/100 g) diets were formulated: a control diet (A0, 0% ABGA) and five experimental diets with varying ABGA inclusion levels (A3: 3%, A6: 6%, A9: 9%, A12: 12%, A15: 15%). The results demonstrated that there were no significant differences (*P > 0.05*) in percentage weight gain (PWG), specific growth rate (SGR), protein efficiency ratio (PER), and feed conversion ratio (FCR) among the experimental groups. Additionally, dietary ABGA did not significantly affect (*P > 0.05*) body carcass composition among different groups. However, amylase activity significantly decreased (*P < 0.05*) in the A12 and A15 fed groups, whereas lipase and protease activities remained insignificant (*P > 0.05*) across all groups. Notably, oxidative stress responses (SOD; superoxide dismutase and CAT; catalase), carbohydrate metabolic enzymes (LDH; lactate dehydrogenase and MDH; malate dehydrogenase), and serum glucose levels increased significantly (*P < 0.05*) with higher ABGA inclusion. Conversely, serum albumin content significantly decreased (*P < 0.05*) in the ABGA-fed groups. There were no significant differences (*P > 0.05*) observed in serum total protein, albumin/globulin (A/G) ratio, aspartate aminotransferase (AST), and alanine aminotransferase (ALT) activities among the experimental groups. Hematological parameters revealed that RBC (red blood cell) count, hemoglobin (Hb) concentration, and packed cell volume (PCV) significantly decreased (*P < 0.05*), while WBC (white blood cell) count significantly (*P < 0.05*) increased with higher dietary ABGA inclusion. In conclusion, the inclusion of dietary ABGA up to 15% did not impair nutrient utilization and supported normal growth performance in *C. catla* fingerlings. However, higher inclusion levels may have a detrimental effect on their growth, nutrient utilization, and physio-metabolic responses.

## Introduction

The increasing global population presents a major challenge in maintaining a stable and nutritious food supply. Aquaculture has emerged as a sustainable solution, providing a controlled environment for the production of nutritious food sources^1–5^. As one of the fastest-growing sectors in food production, aquaculture plays an essential role in enhancing global food security^6,7^. In 2020, aquaculture accounted for approximately 178 million metric tonnes (MMT) of global fish supply^8^. India’s total fish production in 2021-22 reached 16.24 MMT^9^. Fish serves as a vital food source while also contributing significantly to income generation, employment, and recreational activities^4^. Fish serves as an important source of nutrients for addressing global health challenges^10^. Its affordability and rich protein content make it a vital source of nutrition^10^. This has increased dietary acceptance of fish in developed and developing countries^11^. India relies mainly on freshwater fish farming to tackle rising fish demand^12^. Choosing high-demand species is crucial for achieving a greater return on investment for fish farmers^13^. *Catla catla*, a significant cultivable fish species in the Indian subcontinent, contributes 5.6% to global aquaculture production and ranks 6th among the most cultivated aquaculture species^14^. Native to freshwater and primarily farmed in Asia, especially the Indian subcontinent, this crucial species plays a vital role in the region’s food security^15^. As a surface feeder, *C. catla *generally consumes plankton, including phytoplankton and zooplankton, thriving in the riverine systems of northern and central India^16^. Its growth potential and high consumer preference have made it the most crucial freshwater candidate species for culture in India^17^. 

Catla and other Indian major carps (IMC) are the powerhouse behind India’s freshwater aquaculture, contributing around 87% of its production^16^.Catla fingerlings primarily feed on zooplankton, planktonic algae, and vegetable waste, with adults showing a preference for zooplankton^18,19^. Live food also has limitations that will likely hinder its effective utilization^20^. However, the intensification of aquaculture has led to environmental concerns, particularly the accumulation of cyanobacteria biomass, resulting in harmful algal blooms (HABs)^21^. These blooms, characterized by bright green, yellow-brown, and red colours, pose severe water quality problems. Freshwater HABs, typically comprising cyanobacteria, are unsightly and produce foul odours^22,23^. Cyanobacteria release toxic secondary metabolites that adversely affect aquatic animals^24^. HABs erupt when algae and their kin, like dinoflagellates, diatoms, and cyanobacteria, experience explosive growth^25^. The intensification of aquaculture has increased the use of inputs like fertilizers and feed per unit of land, leading to higher waste generation within production systems^26^. This waste contributes to nutrient loading in culture systems and local water bodies, raising sustainability concerns^27,28^. The feed used in aquaculture systems is a significant waste source^28,29^. Decomposed or uneaten feed releases nitrogen (N) and phosphorus (P), which trigger algal bloom production and eutrophication^30^. Blue-green algae (cyanobacteria) are most prevalent due to their hardy nature and adaptability to diverse climatic conditions^31^. Cyanobacterial toxins significantly disrupt ecosystems, persisting and accumulating as they move through the food web^32^. Dating back nearly 3 billion years, cyanobacteria are ancient photosynthetic bacteria (not plants) that played a key role in shaping Earth’s atmosphere^33–35^. Cyanotoxins can cause cytotoxic and genotoxic effects in teleost fishes by generating reactive oxygen species (ROS) and down-regulating antioxidant biomarkers, leading to oxidative stress, genotoxicity, cytotoxicity, and apoptosis^32^. Microcystin-LR, a toxin produced by cyanobacteria, generates excessive reactive oxygen species to exert its toxic effects^36^. Anatoxin-a (ANTX-a), a toxin found in freshwater algae blooms worldwide, poses a growing threat to consumers^37^. Multiple types of cyanobacteria, including Anabaena and Aphanizomenon, churn out this neurotoxin^38–44^. This neurotoxin bioaccumulates in aquatic life, harming them and the ecosystem^25,45^.???

The present study aims to evaluate the effects of increasing dietary levels of the cyanobacterium Anabaena on the growth performance and physio-metabolic responses of the economically important freshwater species *Catla catla*. This research provides insights into the impacts of cyanobacterial blooms, often a consequence of eutrophication, and explores the potential use of eutrophic algal biomass as a sustainable dietary supplement in aquaculture. Such practices are critical for enhancing production efficiency in response to rising protein demand and pressures on aquatic ecosystems.

**Table 1:** Physico-chemical parameters of water in different experimental tanks during the experimental period of 60 days.

Treatments	Temperature (^0^C)	pH	Dissolved oxygen (mg/L)	Ammonia (mg/L)	Nitrite (mg/L)	Nitrate (mg/L)	Free CO_2_ (mg/L)
A0	27–30	7.5–8.4	6.67 ± 0.12	0.029 ± 0.03	0-0.004	0-0.04	ND
A3	28–30	7.5–8.4	6.60 ± 0.16	0.01 ± 0.001	0-0.003	0-0.03	ND
A6	27–29.5	7.6–8.5	6.87 ± 0.19	0.02 ± 0.002	0-0.004	0-0.04	ND
A9	27.5–30	7.8–8.3	6.93 ± 0.15	0.01 ± 0.002	0-0.005	0-0.06	ND
A12	28-30.5	7.8–8.5	6.56 ± 0.19	0.02 ± 0.02	0-0.006	0-0.07	ND
A15	27-30.4	7.6–8.3	6.67 ± 0.12	0.029 ± 0.03	0-0.006	0-0.07	ND

ND- not detected.

## Results

### Physico-chemical parameters of water

The average range of physicochemical parameters of the water studied, including temperature (°C), dissolved oxygen (mg/L), pH, nitrite (mg/L), nitrate (mg/L), free CO2 (mg/L), and ammonia (mg/L), is presented in Table 1.

**Table 2:** Proximate composition of Anabaena powder (% dry matter basis).

Variable	Anabaena powder
Dry matter	92.32 ± 0.06
Crude protein	11.72 ± 0.22
Ether extract	2.91 ± 0.05
Crude fibre	17.72 ± 0.34
Nitrogen free extract	52.46 ± 0.22
Total ash	15.19 ± 0.07

All values are expressed as percentage Mean ± SE (n=3).

3

### Proximate composition of the Anabaena powder

The proximate composition of the Anabaena powder is given in Table 2. The Anabaena powder was from outdoor mass culture, not pure culture.

**Table 3:** The composition and proximate analysis of experimental diets.

Ingredients composition (g/kg)	A0	A3	A6	A9	A12	A15	
Fish meal	200	200	200	200	200	200	
^1^SBM	150	150	150	150	150	150	
^2^MOC	150	150	160	170	180	190	
^3^DORB	170	170	160	150	140	130	
Wheat flour	100	100	100	100	100	100	
Maize flour	150	120	90	60	30	0	
Fish oil	10	10	10	10	10	10	
Soybean oil	20	20	20	20	20	20	
^4^Vitamin min. mix.	30	30	30	30	30	30	
Choline chloride	2	2	2	2	2	2	
Vitamin C	2.5	2.5	2.5	2.5	2.5	2.5	
^5^CMC	15	15	15	15	15	15	
Betaine	0.5	0.5	0.5	0.5	0.5	0.5	
Anabaena powder	0	30	60	90	120	150	
Total	1000	1000	1000	1000	1000	1000	
Proximate composition (% dry matter basis)							
Crude protein	30.88	30.20	30.35	30.98	30.39	30.85	
Ether extract	6.43	6.54	6.46	6.42	6.41	6.43	
Crude fiber	8.59	8.44	8.65	8.41	8.52	8.57	
Nitrogen free extract	47.81	48.58	48.2	47.79	48.39	47.87	
Total ash	6.29	6.24	6.34	6.40	6.29	6.33	
Digestible energy (kcal/100g)	378.25	379.61	377.86	378.31	378.25	378.03	

^1^SBM- Soybean meal; ^2^MOC- Mustard oil cake; ^3^DORB- De-oiled rice bran; ^4^Composition of the Vitamin-Mineral Mixture (per kg)- Vitamin A: 5,500,000 IU; Vitamin D3: 1,100,000 IU; Vitamin B2: 2,000 mg; Vitamin E: 750 mg; Vitamin K: 1,000 mg; Ascorbic Acid: 2,500 mg; Vitamin B6: 1,000 mg; Vitamin B12: 6 mcg; Calcium Pantothenate: 2,500 mg; Nicotinamide: 10 g; Choline Chloride: 150 g; Manganese (Mn): 27,000 mg; Iodine (I): 1,000 mg; Iron (Fe): 7,500 mg; Zinc (Zn): 5,000 mg; Copper (Cu): 2,000 mg; Cobalt (Co): 450 mg; Selenium (Se): 125 mg; ^5^CMC-Carboxymethyl cellulose; Digestible energy: (kcal/100g) = (%CP x 4) + (%EE x 9) + (%TC x 4)^46^.

### Proximate analysis of the experimental diets (% dry matter basis)

The proximate composition of the experimental diets is summarized in Table 3. The dry matter (DM) content ranged from 91.90 to 92.00%, crude protein (CP) from 30.20 to 30.98%, ether extract (EE) from 6.41 to 6.54%, crude fibre (CF) from 8.41 to 8.65%, nitrogen-free extract (NFE) from 47.79 to 48.58%, and total ash content (TA) from 6.24 to 6.40%. The diets’ estimated digestible energy (DE) values were between 377.86 and 379.17 kcal/100 g of feed.

### Whole body composition of *C. catla* fingerlings (% wet weight basis)

Table 4 details the biochemical composition of *C. catla* fingerlings, encompassing moisture, CP, EE, TC, and TA. The analysis indicated that the whole-body composition parameters remained consistent across different dietary treatments, showing no significant differences (*P > 0.05*).

**Table 4:** Whole body chemical composition of *C. catla* fingerlings of different experimental groups (% wet weight basis).

Treatments	Moisture	Crude Protein	Crude lipid	Total ash	Total carbohydrate
A0	76.99 ± 0.66	16.48 ± 0.39	2.44 ± 0.09	2.79 ± 0.05	1.30 ± 0.18
A3	77.02 ± 0.45	16.41 ± 0.36	2.39 ± 0.06	2.78 ± 0.13	1.40 ± 0.31
A6	77.09 ± 0.21	16.26 ± 0.60	2.47 ± 0.12	2.70 ± 0.04	1.25 ± 0.24
A9	77.16 ± 0.29	15.99 ± 0.30	2.43 ± 0.15	2.77 ± 0.12	1.45 ± 0.38
A12	76.94 ± 0.06	16.66 ± 0.21	2.45 ± 0.17	2.88 ± 0.19	1.47 ± 0.13
A15	77.12 ± 0.25	16.26 ± 0.74	2.47 ± 0.08	2.75 ± 0.16	1.40 ± 0.30
***P-value***	*0.974*	*0.946*	*0.340*	*0.846*	*0.869*

Data expressed as Mean ± SE (*n* = 3).

### Growth performance and nutrient utilization indices

Table 5 illustrates growth performance and nutrient utilization parameters subjected to various experimental diets. Percentage weight gain (PWG) did not differ significantly (*P > 0.05*) among the treatment groups. Similarly, the specific growth rate (SGR) did not exhibit significant variation (*P > 0.05*) across the experimental groups. Additionally, the feed conversion ratio (FCR), feed efficiency ratio (FER), and protein efficiency ratio (PER) analysis also showed no significant differences (*P > 0.05*) across the dietary treatments. These findings suggest that the different diets did not significantly impact the growth performance or nutrient utilization of *C. catla* fingerlings.

**Table 5:** Growth performances of *C. catla* fed different experimental diets for 60 days.

Treatments	^1^PWG	^2^SGR	^3^FCR	^4^FER	^5^PER
A0	105.76 ± 2.75	1.20 ± 0.02	1.70 ± 0.04	0.59 ± 0.02	1.96 ± 0.05
A3	104.50 ± 4.50	1.19 ± 0.03	1.73 ± 0.09	0.58 ± 0.03	1.95 ± 0.08
A6	106.00 ± 5.00	1.21 ± 0.03	1.71 ± 0.08	0.60 ± 0.03	1.97 ± 0.08
A9	103.00 ± 5.50	1.18 ± 0.04	1.75 ± 0.10	0.57 ± 0.03	1.94 ± 0.09
A12	104.00 ± 6.00	1.19 ± 0.04	1.72 ± 0.09	0.59 ± 0.03	1.96 ± 0.10
A15	103.50 ± 4.50	1.18 ± 0.03	1.74 ± 0.08	0.58 ± 0.02	1.94 ± 0.08
***P-Value***	*0.339*	*0.341*	*0.354*	*0.339*	*0.339*

All values are expressed as Mean ± SE (n=3).

^1^PWG - Percentage weight gain; ^2^SGR - Specific growth rate; ^3^FCR - Feed conversion ratio; ^4^FER - Feed efficiency ratio, ^5^PER - Protein efficiency ratio.

### Apparent digestibility coefficient of the experimental diets

Table 6 shows the apparent dry matter digestibility coefficient (ADMDC) values for *C. catla* fingerlings among all treatment groups (A0, A3, A6, A9, A12, and A15). The ADMDC values did not differ significantly (*P > 0.05*) from the control group (A0).

**Table 6:** Apparent digestibility coefficient (ADC) dry matter and protein of experimental diets.

Treatments	^1^ADMDC (%)	^2^ACPDC (%)
A0	65.22 ± 0.06	84.09 ± 0.09
A3	66.08 ± 0.32	83.78 ± 0.15
A6	65.48 ± 0.27	83.90 ± 0.31
A9	66.54 ± 0.42	83.79 ± 0.55
A12	65.90 ± 0.24	83.79 ± 0.19
A15	65.84 ± 0.31	83.44 ± 0.48
***P- Value***	*0.121*	*0.446*

All values are expressed as Mean ± SE (*n* = 3).

^1^ADMDC- Apparent dry matter digestibility coefficient.

^2^ACPDC- Apparent crude protein digestibility coefficient.

### Digestive enzyme activities

Table 7 shows the recorded activities of digestive enzymes, including protease, amylase, and lipase, in the intestines of *C. catla* fingerlings across various experimental groups. The specific activities of intestinal protease in *C. catla* did not exhibit significant differences (*P > 0.05*) among the different dietary treatment groups. In contrast, the intestinal amylase activities varied significantly (*P < 0.05*) among the groups, with the highest levels observed in the control (A0) and A3-fed groups. Similarly, the intestinal lipase activities showed significant variation (*P < 0.05*) across all dietary treatment groups.

**Table 7:** Digestive enzyme activities in the intestine of *C. catla* fingerlings fed with different experimental diets.

Treatments	^1^Protease	^2^Amylase	^3^Lipase
A0	103.12 ± 1.50	1.98 ± 0.05 ^c^	0.47 ± 0.01 ^c^
A3	102.63 ± 1.49	1.64 ± 0.07 ^bc^	0.23 ± 0.02 ^bc^
A6	102.73 ± 1.19	1.39 ± 0.03 ^a^	0.27 ± 0.01
A9	102.46 ± 1.16	1.55 ± 0.03 ^b^	0.12 ± 0.00 ^a^
A12	102.96 ± 1.32	1.37 ± 0.03 ^a^	0.10 ± 0.01 ^a^
A15	102.40 ± 1.87	1.53 ± 0.02 ^b^	0.18 ± 0.01 ^b^
***P-Value***	*0.917*	*< 0.001*	*0.005*

All values are expressed as Mean ± SE (*n*=3). Mean values in the same column with different superscripts differ significantly (*P < *0.05).

^1^Protease activity is expressed in micromole of tyrosine released/min/mg protein at 37 °C.

^2^Amylase activity is expressed in micromole of maltose released /min/mg protein at 37 °C.

^3^Lipase activity is expressed in units/hour/mg protein at 37 °C.

### Metabolic enzymes

#### Protein metabolic response

Table 8 presents the aspartate aminotransferase (AST) activities in the liver and muscle of *C. catla* fingerlings subjected to various experimental diets. Notably, there were no significant differences (*P > 0.05*) in AST activity between the control (A0) and other experimental groups in both muscle and liver tissues. Similarly, alanine aminotransferase (ALT) activities in the liver and muscle of *C. catla* fingerlings across different dietary treatments showed no significant (*P > 0.05*) variation among the dietary treatment groups.

**Table 8:** Activities of protein metabolic enzymes in *C. catla* fed with different experimental diets.

^1^Treatments	^1^ALT		^2^AST	
	Muscle	Liver	Muscle	Liver
A0	9.50 ± 0.30	5.69 ± 0.05	12.98 ± 0.13	3.19 ± 0.11
A3	9.46 ± 0.06	5.55 ± 0.10	12.00 ± 0.11	3.25 ± 0.19
A6	9.62 ± 0.12	5.66 ± 0.09	12.11 ± 0.15	3.34 ± 0.25
A9	9.60 ± 0.10	5.60 ± 0.03	12.01 ± 0.05	3.17 ± 0.09
A12	9.56 ± 0.11	5.46 ± 0.16	12.04 ± 0.10	3.29 ± 0.01
A15	9.49 ± 0.14	5.52 ± 0.18	12.93 ± 0.10	3.37 ± 0.31
***P-Value***	*0.993*	*0.522*	*0.321*	*0.521*

Data expressed as Mean ± SE *n *= 3.

^1^ALT - Alanine aminotransferase, specific activities expressed as nanomoles of sodium pyruvate released/min/mg protein at 37^0^C.

^2^AST - Aspartate aminotransferase, specific activities expressed as nanomoles of oxaloacetate released/min/mg protein at 37^0^C.

#### Carbohydrate metabolic response

The lactate dehydrogenase (LDH) activities in the liver and muscle of *C. catla* across various experimental groups are presented in Table 9. Notably, there was no significant (*P > 0.05*) difference in liver LDH activity between the control group and the dietary treatments in muscle. However, a significant increase (*P < 0.05*) was observed in fish fed an Anabaena-based diet in the liver. The malate dehydrogenase (MDH) activities in the liver and muscle of *C. catla* fingerlings showed enhanced MDH activities observed in the muscle and liver fed with various dietary inclusions of Anabaena powder.

**Table 9:** Activities of carbohydrate metabolic enzymes in *C. catla* fingerlings fed with different experimental diets for 60 days.

Treatments	^1^LDH		^2^MDH	
	Liver	Muscle	Liver	Muscle
A0	4.27 ± 0.44 ^a^	10.33 ± 0.36	0.47 ± 0.02 ^a^	1.83 ± 0.02 ^a^
A3	9.77 ± 0.42 ^bc^	12.90 ± 0.72	0.87 ± 0.01 ^c^	2.12 ± 0.13 ^ab^
A6	8.31 ± 0.07 ^b^	8.20 ± 1.01	0.64 ± 0.04 ^b^	2.24 ± 0.01 ^bc^
A9	9.22 ± 0.50 ^b^	11.49 ± 1.03	0.61 ± 0.01 ^b^	2.55 ± 0.17 ^c^
A12	9.90 ± 0.73 ^bc^	11.14 ± 1.41	0.67 ± 0.06 ^b^	2.37 ± 0.17 ^bc^
A15	11.24 ± 0.67 ^c^	11.14 ± 0.27	0.62 ± 0.03 ^b^	2.31 ± 0.09 ^bc^
***P-Value***	*< 0.001*	*0.045*	*< 0.001*	*0.018*

Data expressed as Mean ± SE *n *= 3; Mean values in the same column with different superscripts differ significantly (*P *< 0.05).

^1^LDH - Lactate dehydrogenase, activity expressed in Unit/mg protein/min at 37°C.

^2^MDH - Malate dehydrogenase, activity expressed in Unit/mg protein/min at 37°C.

### Oxidative stress response

**Fig. 1:**
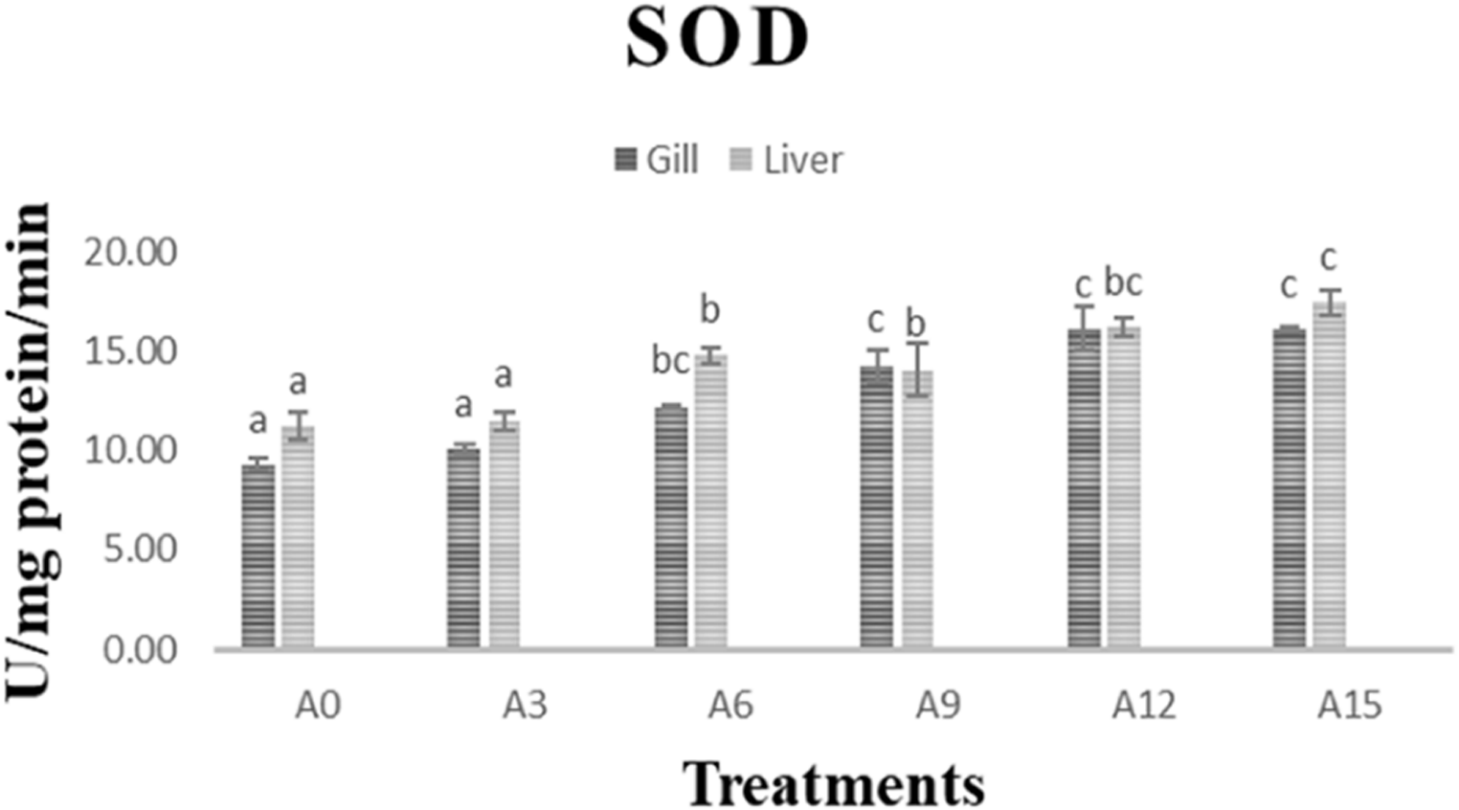
Illustrate the oxidative stress activity in *C. catla* fingerlings subjected to various experimental diets.

**Fig. 2:**
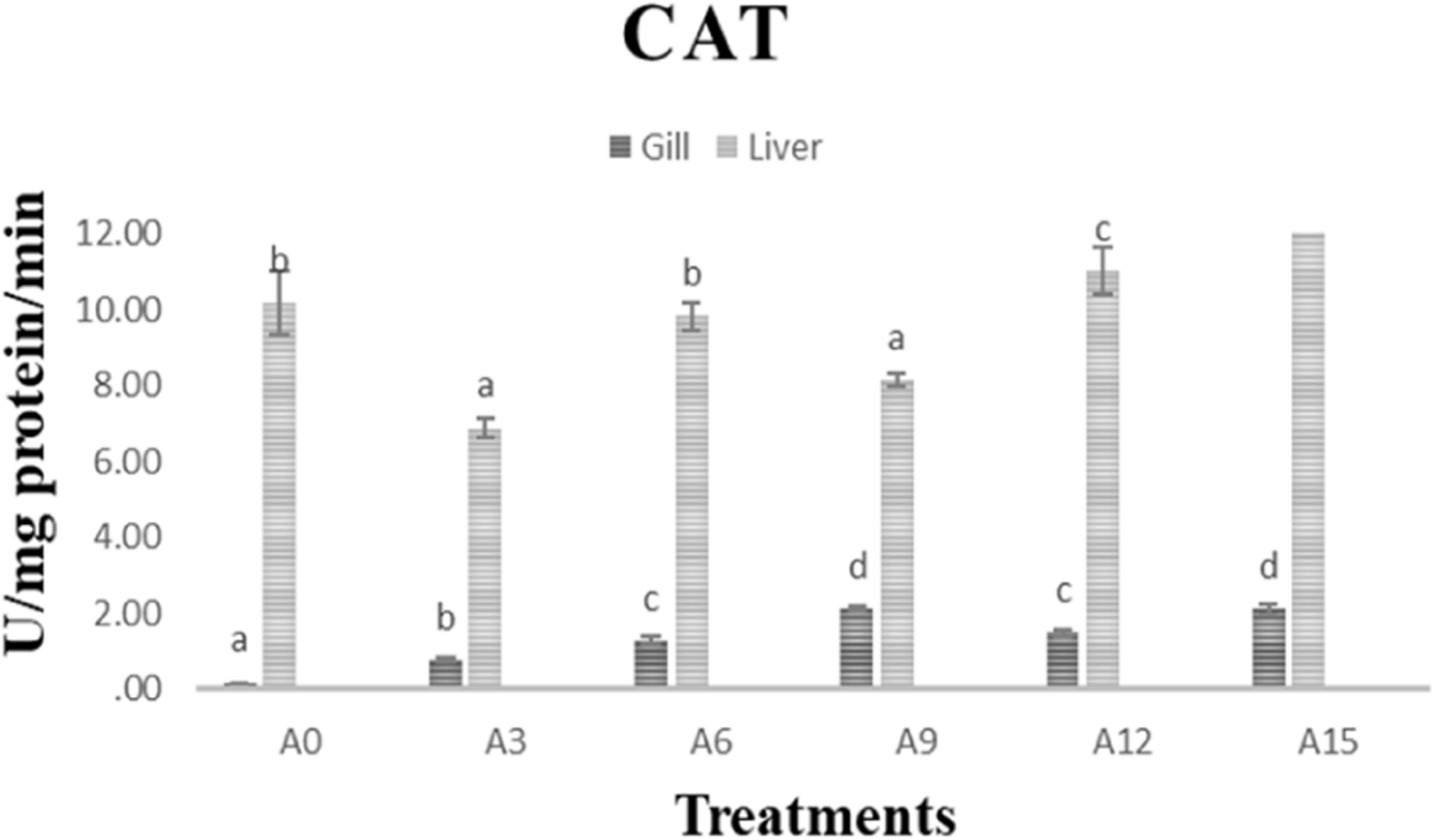
Illustrate the oxidative stress activity in *C. catla* fingerlings subjected to various experimental diets.

Figure 1 illustrates the superoxide dismutase (SOD) activities in the gills and liver of *C. catla* fingerlings subjected to various experimental diets. The results indicate a significant increase (*P < 0.05*) in SOD activities in both tissues for the groups supplemented with Anabaena. Similarly, Fig. 2 depicts the catalase (CAT) activity in the gills and liver, and notably, the Anabaena supplemented groups exhibited significantly higher (*P < 0.05*) CAT activities in both the liver and gills.

### Serum parameters

Table 10 summarizes the serum concentrations of glucose, total protein, albumin, globulin, and the albumin-to-globulin (A/G) ratio, along with the results of the Nitroblue Tetrazolium (NBT) assay for the experimental groups. Serum glucose levels differed significantly (*P < 0.05*), showing an increase with the inclusion of dietary Anabaena; the A15 group exhibited the highest levels (140.41 ± 3.34), while the control group (A0) exhibited the lowest (38.65 ± 0.64). Furthermore, serum albumin levels also varied significantly (*P < 0.05*), with the control group (A0) having higher levels compared to the other groups. There were no significant differences (*P > 0.05*) in total protein concentrations, globulin levels, or the A/G ratio between the control and treatment groups. NBT activity in *C. catla* fingerlings showed no significant (*P > 0.05*) variation across the different diets.

**Table 10:** Serum total protein, albumin (A), globulin (G), a: G ratio, and glucose content of the different experimental groups.

Treatments	Total protein	Albumin	Globulin	A/G ratio	Glucose	^1^NBT
A0	5.18 ± 0.2	2.87 ± 0.1 ^c^	2.31 ± 0.01	1.25 ± 0.01	38.65 ± 0.64 ^a^	0.43 ± 0.2
A3	5.77 ± 0.3	2.69 ± 0.03 ^b^	3.07 ± 0.02	0.89 ± 0.05	48.49 ± 4.82 ^b^	0.43 ± 0.7
A6	5.14 ± 0.3	2.47 ± 0.01 ^a^	2.66 ± 0.02	0.96 ± 0.01	49.89 ± 3.87 ^b^	0.38 ± 0.9
A9	5.57 ± 0.2	2.40 ± 0.01 ^a^	3.17 ± 0.08	0.76 ± 0.05	64.86 ± 1.82 ^c^	0.37 ± 0.5
A12	4.98 ± 0.3	2.42 ± 0.01 ^a^	2.56 ± 0.03	0.99 ± 0.03	93.42 ± 1.17 ^d^	0.49 ± 0.3
A15	5.65 ± 0.3	2.34 ± 0.01 ^a^	3.31 ± 0.05	0.72 ± 0.03	140.41 ± 3.34 ^e^	0.36 ± 0.1
***P-Value***	*0.372*	*0.001*	*0.218*	*0.072*	*< 0.001*	*0.134*

Data are expressed as Mean ± SE. *n* =3; Mean values in the same column with different superscripts differ significantly (*P < *0.05).

^1^NBT- Nitroblue tetrazolium.

### Haematological parameters

Table 11 details the haematological parameters of *C. catla* fingerlings subjected to various dietary treatments. Significant differences (*P < 0.05*) were observed in haemoglobin concentration (Hb), total erythrocyte count (RBC), and packed cell volume (PCV) among the different diet groups, with the control group exhibiting higher Hb and PCV levels. In contrast, total leucocyte count (WBC) did not differ significantly (*P > 0.05*) across the groups.

**Table 11:** Haematological parameters of *C. catla* fingerlings fed different experimental diets.

Treatments	^1^Hb (g/dl)	^2^RBC (10^6^ cells mm^3^)	^3^WBC (10^3^ cells mm)	^4^PCV (%)
A0	6.37 ± 0.26 ^c^	2.03 ± 0.09 ^c^	38.00 ± 2.31	19.0 ± 0.78 ^c^
A3	5.43 ± 0.09 ^b^	1.83 ± 0.15 ^bc^	30.50 ± 0.87	15.6 ± 0.35 ^b^
A6	5.50 ± 0.23 ^b^	1.80 ± 0.06 ^bc^	39.83 ± 7.53	16.6 ± 0.70 ^b^
A9	4.20 ± 0.17 ^a^	1.40 ± 0.06 ^a^	34.33 ± 7.13	12.7 ± 0.52 ^a^
A12	4.13 ± 0.15 ^a^	1.43 ± 0.03 ^a^	43.33 ± 4.84	12.9 ± 0.25 ^a^
A15	4.70 ± 0.12 ^a^	1.63 ± 0.09 ^ab^	53.00 ± 5.86	14.9 ± 0.95 ^b^
***P-Value***	*< 0.001*	*< 0.001*	*0.129*	*< 0.001*

Data expressed as Mean ± SE, (*n *= 3). Mean values in the same column with different superscripts differ significantly(*P < *0.05).

^1^Hb - Haemoglobin; ^2^RBC- Red blood corpuscles; ^3^WBC - White blood corpuscles; ^4^PVC -Packed cell volume.

## Discussion

The present study explores the multifaceted effects of incorporating Anabaena, a cyanobacterial genus, into the diet of *Catla catla*fingerlings, focusing on key physiological and biochemical parameters. The investigation aims to evaluate the impact of this dietary modification on nutrient utilization, growth performance, and overall health status of the fish. Fish are sensitive to changes in their aquatic environment, so maintaining optimal water quality in aquaculture systems is essential. Ensuring stable water parameters is crucial for maximizing their survival, growth, and overall health^47^. In this study, water quality parameters were meticulously maintained within optimal ranges suitable for freshwater fish culture. Given that fish are poikilothermic organisms, their metabolic rates are significantly influenced by temperature^48^. Optimal metabolic activity and maximum yield for *C. catla*are achieved within a specific temperature range^19^. In this study, observed temperatures aligned with the optimal range. Moreover, pH levels across all experimental groups were within the recommended limits^49^. The experimental tanks’ DO levels were maintained within the desired concentrations, aligning with the recommended values for optimal fish health. In addition, ammonia concentrations in the water were controlled within the safe range^19^. By maintaining these physico-chemical characteristics, the study establishes a stable environment that supports the health and growth of *C. catla*, highlighting the importance of water quality management in aquaculture.

The experimental diets were formulated to be iso-nitrogenous (30% protein) and iso-lipidic (6% lipid), based on the optimal levels recommended for Indian major carps (IMC) fingerlings^50^. The test diets provided usable energy within a range consistent with previous findings suggesting a digestible energy requirement of 350–400 kcal/100 g for Catla fingerlings^51^.

Dietary supplementation with Anabaena had no significant effect on the proximate composition of the fish, including moisture, crude protein (CP), ether extract (EE), total carbohydrates (TC), and ash content. This outcome is consistent with previous studies on Nile tilapia fingerlings, where Anabaena supplementation similarly showed no substantial influence on body composition^52^. Conversely, Goldfish fed with lyophilized blue-green algae (BGA) powder exhibited significant changes in body composition after 16 weeks, showing significant reductions in CP, EE, and total ash levels^53^. The protein content of IMCs typically ranges from 16 to 19%^54^. The fish-fed diets with or without Anabaena for 60 days exhibited no significant difference in body protein content, indicating that the inclusion of Anabaena had minimal impact on protein levels.

Growth parameters, including weight gain (WG), percent weight gain (%WG), specific growth rate (SGR), feed efficiency ratio (FER), and protein efficiency ratio (PER), were assessed, and results indicated that Anabaena supplementation did not significantly influence any of these growth metrics. This finding is consistent with a study that showed no significant changes in growth among female Nile tilapia-fed diets containing different levels of dried cyanobacterial biomass and *Arthrospira*(cyanobacteria) over a 28-day period^55^. Adding up to 15% Anabaena to the fish diet had no significant effect on their growth compared to the control group.

A crucial factor in selecting ingredients for fish feed is their digestibility by the targeted fish species^56^. In this study, the apparent crude protein digestibility coefficient (ACPDC), apparent lipid digestibility (ALDC), and apparent dry matter digestibility coefficient (ADMDC) of Anabaena-containing diets did not significantly differ from the control diet. Our results support that dietary Anabaena had no significant impact on liver enzymes in Nile tilapia^52^.

The breakdown of complex nutrients into smaller, absorbable components in an animal’s digestive system is highly dependent on the presence and accessibility of specific enzymes^20^. This study found that *C. catla*fingerlings fed different diets had varying levels of amylase activity in their intestines. Despite variations in dietary regimens, no significant differences were observed in lipase and protease activities. Certain cyanobacteria exhibit a multifaceted impact on the gastrointestinal tract. These microorganisms can disrupt the intestinal barrier function, alter the composition of gut microbiota, and interfere with the production of digestive enzymes and inflammatory signaling molecules^57^. Consequently, these effects may influence the activation of immune genes within the intestinal mucosa^58^. Silver and bighead carp can digest Microcystis, albeit with poor digestibility^59^. A study found that silver carp weren’t very good at digesting or using *M. aeruginosa*as a food source^60^. Microcystis, due to its low digestibility, passes through the digestive system of the roach (*Rutilus rutilus*) undigested and undergoes exponential growth following excretion^61^.

Aspartate aminotransferase (AST) and alanine aminotransferase (ALT) are essential enzymes moving nitrogen groups between different amino acids within the body^62^. AST and ALT catalyze transamination reactions, transferring an amino group between amino acids and α-keto acids^63^. AST exchanges the amino group of aspartates with the keto group of α-ketoglutarate, yielding oxaloacetate and glutamate. ALT performs a similar reaction, replacing aspartate with alanine, producing glutamate and pyruvate^64^. These pyruvate and oxaloacetate molecules are essential for synthesizing non-essential amino acids such as alanine, asparagine, and glutamine, which are important for animal protein synthesis and growth. Additionally, amino acids can undergo deamination to generate intermediates for the tricarboxylic acid (TCA) cycle, thereby contributing to ATP production and energy metabolism^65^. The present study assessed ALT and AST activities in the muscle and liver, revealing no significant differences among the dietary treatments with Anabaena supplementation. While microcystins have been reported to impact fish liver function^66–68^, our findings did not indicate significant alterations in ALT and AST activities due to Anabaena supplementation. Interestingly, some previous studies showed that the levels of glutamate pyruvate transaminase (GPT) and glutamate oxaloacetate transaminase (GOT) in the blood plasma of common carp (*Cyprinus carpio L.*) increased after intraperitoneal injections of MC-LR, indicating that cyanotoxins may adversely affect liver function^67,69^.

Lactate dehydrogenase (LDH) acts as the final enzyme in the glycolytic pathway, catalyzing the conversion of lactate to pyruvate in the presence of the coenzyme NADH, which is simultaneously oxidized to NAD +^70^. This reaction is crucial for sustaining glycolysis by regenerating NAD+. In aerobic conditions, pyruvate proceeds into the Krebs cycle; however, under anaerobic conditions, it is reduced back to lactate^71^. In the present study, elevating the inclusion level of Anabaena significantly enhanced LDH activities in both the liver and muscle. This increase is likely due to elevated lactate production, which serves as fish’s preferred substrate for gluconeogenesis during anaerobic metabolism^72^. Generally, LDH activity increases under stress conditions such as temperature stress^73^, starvation stress^74^, and confinement stress^75^. Exposure to antimetabolites stimulates liver cells to neutralize and eliminate them^76^. This mechanism entails heightened aryl hydrocarbon hydroxylase (AHH) activity, an enzymatic system crucial for detoxifying hazardous hydrocarbons that rely on molecular oxygen for their processing^77^. As a result, there is a competition for oxygen between detoxification processes and the electron transport chain (ETC.), which can lead to increased activity of LDH, an enzyme in the liver^78^. Malate dehydrogenase (MDH), a key enzyme in the energy-generating citric acid cycle, can switch malate into oxaloacetate and back again^79^. The activity of malate dehydrogenase (MDH), an enzyme derived from amino acids, is expected to increase during gluconeogenesis. Our study observed a significant elevation in muscle MDH activity as the proportion of Anabaena in the diet increased.

Organisms utilize diverse defence mechanisms against reactive oxygen species (ROS), such as antioxidant enzymes like superoxide dismutase (SOD) and catalase (CAT)^80^. Oxidative stress arises when pro-oxidant factors overpower antioxidant defences, resulting in insufficient removal of ROS^81^. SOD facilitates the dismutation of superoxide ions into oxygen and hydrogen peroxide, whereas CAT decomposes hydrogen peroxide into water and oxygen^82^. These enzymes are pivotal in serving as antioxidant defences in organisms subjected to oxygen exposure. In instances of heightened oxidative stress, the levels of SOD and CAT generally increase. Loach fish fed with *Microcystis*mixed with commercial eel food powder exhibited significantly increased activities of antioxidant enzymes (SOD, CAT)^83^. A simultaneous induction response in SOD and CAT activities was noted after exposure to contaminants^84^, while enhanced SOD and CAT activities were observed in MC-LR-induced common carp hepatocytes^84,85^. Similarly, increased activity of antioxidant enzymes (SOD, CAT, GPx, GR) was observed in Tilapia fish subjected to cyanobacterial cells^86^. Our findings corroborate previous studies, demonstrating that Anabaena supplementation significantly increased superoxide dismutase (SOD) activity in fish compared to the control. Furthermore, higher levels of Anabaena inclusion (12% and 15%) were correlated with elevated catalase (CAT) activity, suggesting increased oxidative stress at these inclusion rates. This suggests that antioxidant enzymes are triggered as a defensive mechanism against *Microcystis*toxins in Tilapia^86^.

Glucose is a crucial animal energy source and a stress indicator influenced by physical factors^87–89^. During stress, catecholamine secretion increases, leading to glycogen breakdown and elevated blood glucose levels^90^. Blood glucose levels are controlled by the absorption of glucose in the intestines, the production of glucose in the liver, and the uptake of glucose by tissues via processes such as hepatic glycogenolysis, glycolysis, and gluconeogenesis^91^. The present study found that dietary supplementation with 15% Anabaena increased blood glucose levels, suggesting physiological stress in the fish. Furthermore, serum protein levels were evaluated, as they serve as important biomarkers of non-specific immunity and overall fish health^20,92^. Immune stimulants can boost protein synthesis, producing immune-related molecules such as immunoglobulin, complement, lysozyme, and anti-proteases, which play roles in immunity^93^. The addition of Anabaena in this study did not result in any changes to serum protein levels. Serum albumins and globulins are vital components that contribute significantly to immune responses^94^. The liver plays a crucial role in synthesizing albumins, which help maintain vascular fluid volume, and globulins, especially gamma globulins, are vital for immune function^95^. Anabaena supplementation significantly lowered serum albumin levels. While serum globulin levels and the A: G ratio remained relatively unchanged, haematological parameters were adversely affected, with decreases in RBC count, Hb, and PCV, and an increase in WBC count. These findings suggest a detrimental effect of Anabaena inclusion on overall protein status and haematopoiesis. Nile Tilapia-fed diets containing Anabaena exhibited comparable results to those reported in previous studies, with a negative impact observed at higher inclusion levels^96^. The Nitroblue tetrazolium (NBT) assay revealed the lowest respiratory burst activity in the A15 group and the highest in the control group. Plant ingredients might reduce RBC and Hb concentrations^97^. Reduced protein metabolism could decrease RBC synthesis, lowering Hb content and NBT-induced immune response^97^. The elevated presence of toxic metabolites in Anabaena-based diets was likely a contributing factor to the observed changes in haematological and immune parameters in the fish.

## Materials and methods

### Experimental site

The 60-day feeding trial was conducted at the ICAR-Central Institute of Fisheries Education, Kolkata Centre. Subsequently, laboratory analyses were performed in the Fish Nutrition, Biochemistry, and Physiology Laboratory at the same institute.

### Procurement and acclimatization of experimental animals

Specimens of *C. catla* were sourced from a fish seed farm located in Naihati, West Bengal, India, and transported to the ICAR-CIFE Kolkata Centre in oxygenated plastic bags to ensure their viability. Upon arrival, the fish were promptly placed into three 1000 L FRP tanks and allowed to rest overnight to recover from transportation stress. To reduce handling-induced stress, they were treated with a mild solution of salt and vitamin C in aerated tanks before undergoing a 15-day acclimatization period on a standard diet. Following acclimatization, the experiment commenced with their transfer to the designated rearing tanks.

### Experimental units

For the feeding trial, 18 rectangular FRP tanks (800 L capacity each) were used. The tanks were covered with mosquito netting to prevent fish escape. Before the experiment, the tanks were disinfected with a 4-ppm potassium permanganate (KMnO₄) solution overnight. After disinfection, they were thoroughly washed and sun-dried for 12 h. The tanks were then filled with 550 L of dechlorinated bore-well water, and continuous aeration was provided using air stones.

### Experimental design and feeding

Two hundred seventy *C. catla* fingerlings (initial average weight 9.45 ± 0.15 g) were divided into six experimental groups (in triplicate). Six diets containing varying levels of Anabaena blue-green algae (0%, 3%, 6%, 9%, 12%, and 15%) were prepared, with a stocking density of 15 fish/ tank in a completely randomized design (CRD). The fish were fed to apparent satiation twice daily (7.30 h, 17.00 h). Daily tank maintenance included cleaning and replacing some portion of water using a siphon.

### Physico-chemical parameters of water

The water temperature was checked twice daily using a Master test kit water thermometer (TSi15, UK). The MERCK dissolved oxygen (DO) meter (Germany) was used to measure DO. Daily, the pH of the water was assessed using a Master test kit pH solution (XL, UK). Total ammonia nitrogen concentrations were measured using an ammonia-nitrite test kit (ICAR-CIFE, Mumbai, India), and the results were expressed in mg/L. Nitrite-N concentrations were measured every three days using the same test kit, and the values were reported in mg/L. Nitrate-N concentrations were reported in mg/L using the same kit. Dissolved free carbon dioxide (CO_2_) levels were determined by a titrimetric method^98^.

### Formulation and preparation of the experimental diets

Six experimental diets were prepared (Table 1), with varying levels of Anabaena blue-green algae at 0%, 3%, 6%, 9%, 12%, and 15% (designated as A0, A3, A6, A9, A12, and A15). These diets were standardized to maintain equal nitrogen and energy content, incorporating fishmeal, soybean meal, mustard oil cake, and Anabaena blue-green algae powder as primary protein sources. Lipids were derived from fish and soybean oil, while carbohydrates were sourced from rice bran, maize, and wheat flour. Choline chloride was used as an attractant, supplemented by a comprehensive vitamin-mineral mix to meet the nutritional needs of the fish. Additional nutrients, including vitamin C, were included as per the dietary requirements. All ingredients were precisely measured and combined in a large plastic container. The mixture was homogenized to form a dough with the desired water content. The dough was steamed in a pressure cooker for 20 min, then cooled. Oils and a vitamin-mineral premix were added and mixed thoroughly. The dough was pelletized (SB, Panchal, Mumbai, India) into uniformly sized (2 mm) pellets. The pellets were air-dried on paper sheets and packaged in airtight polythene bags, each labeled with its respective treatment.

### Proximate analysis of diets and carcass

The proximate composition (moisture, crude protein (CP), ether extract (EE), crude fiber (CF), and total ash of both the experimental diets and whole fish bodies (% dry matter basis) were analyzed^13,99^.The nitrogen-free extract (NFE) for diets and total carbohydrates (TC) in whole fish bodies were calculated using the subtraction method^13^.

The proximate analysis was calculated using the following formulas:

$$\:\text{Moisture}\:\left({\%}\right)=\frac{\text{W}\text{t}.\:\text{o}\text{f}\:\text{t}\text{h}\text{e}\:\text{s}\text{a}\text{m}\text{p}\text{l}\text{e}\:\text{b}\text{e}\text{f}\text{o}\text{r}\text{e}\:\text{d}\text{r}\text{y}\text{i}\text{n}\text{g}\:\left(\text{g}\right)\:-\:\text{W}\text{t}.\:\text{o}\text{f}\:\text{t}\text{h}\text{e}\:\text{s}\text{a}\text{m}\text{p}\text{l}\text{e}\:\text{a}\text{f}\text{t}\text{e}\text{r}\:\text{d}\text{r}\text{y}\text{i}\text{n}\text{g}\:\left(\text{g}\right)}{\text{W}\text{t}.\:\text{o}\text{f}\:\text{t}\text{h}\text{e}\:\text{s}\text{a}\text{m}\text{p}\text{l}\text{e}\:\text{b}\text{e}\text{f}\text{o}\text{r}\text{e}\:\text{d}\text{r}\text{y}\text{i}\text{n}\text{g}\:\left(\text{g}\right)}\text{x}\:\:100$$

$$\:\text{Dry}\:\text{m}\text{a}\text{t}\text{t}\text{e}\text{r}\:\left({\%}\right)\:=\:100\:\:\text{M}\text{o}\text{i}\text{s}\text{t}\text{u}\text{r}\text{e}\:\left({\%}\right)$$

$$\:\text{C}\text{P}\:\left({\%}\right)\:=\:\text{T}\text{o}\text{t}\text{a}\text{l}\:\text{n}\text{i}\text{t}\text{r}\text{o}\text{g}\text{e}\text{n}\:\text{c}\text{o}\text{n}\text{t}\text{e}\text{n}\text{t}\:\left({\%}\right)\:\text{X}\:6.25$$

$$\:\text{E}\text{E}\:\left({\%}\right)=\frac{\text{W}\text{e}\text{i}\text{g}\text{h}\text{t}\:\text{o}\text{f}\:\text{t}\text{h}\text{e}\:\text{f}\text{l}\text{a}\text{s}\text{k}\:\text{p}\text{l}\text{u}\text{s}\:\text{e}\text{x}\text{t}\text{r}\text{a}\text{c}\text{t}\text{e}\text{d}\:\text{l}\text{i}\text{p}\text{i}\text{d}\:\left(\text{g}\right)\:\text{W}\text{e}\text{i}\text{g}\text{h}\text{t}\:\text{o}\text{f}\:\text{t}\text{h}\text{e}\:\:\text{f}\text{l}\text{a}\text{s}\text{k}\:\left(\text{g}\right)}{\text{W}\text{e}\text{i}\text{g}\text{h}\text{t}\:\text{o}\text{f}\:\text{t}\text{h}\text{e}\:\text{d}\text{r}\text{i}\text{e}\text{d}\:\text{s}\text{a}\text{m}\text{p}\text{l}\text{e}\:\left(\text{g}\right)}$$

$$\:\text{C}\text{F}\:\left({\%}\right)=\frac{\text{W}\text{e}\text{i}\text{g}\text{h}\text{t}\:\text{o}\text{f}\:\text{c}\text{r}\text{u}\text{c}\text{i}\text{b}\text{l}\text{e}\:\text{w}\text{i}\text{t}\text{h}\:\text{d}\text{r}\text{y}\:\text{r}\text{e}\text{s}\text{i}\text{d}\text{u}\text{e}-\text{W}\text{e}\text{i}\text{g}\text{h}\text{t}\:\text{o}\text{f}\:\text{c}\text{r}\text{u}\text{c}\text{i}\text{b}\text{l}\text{e}\:\text{w}\text{i}\text{t}\text{h}\:\text{a}\text{s}\text{h}}{\text{W}\text{e}\text{i}\text{g}\text{h}\text{t}\:\text{o}\text{f}\:\text{s}\text{a}\text{m}\text{p}\text{l}\text{e}}\times\:100$$

$$\:\text{T}\text{o}\text{t}\text{a}\text{l}\:\text{a}\text{s}\text{h}\:\left({\%}\right)=\frac{\begin{array}{c}Weight\:of\:the\:ash\:\left(g\right)\end{array}}{\begin{array}{c}Weight\:of\:the\:dry\:sample\end{array}\:\text{t}\text{a}\text{k}\text{e}\text{n}\:\left(\text{g}\right)}\text{x}\:100$$

$$\:\text{N}\text{F}\text{E}\:\left({\%}\right)\:=\:100-\:(\text{C}\text{P}{\%}\:+\:\text{E}\text{E}{\%}\:+\:\text{C}\text{F}\:+\:\text{T}\text{A}{\%})$$

$$\:\text{T}\text{C}\:\left({\%}\right)\:=\:100\:-\:(\text{C}\text{P}{\%}\:+\:\text{E}\text{E}{\%}\:+\:\text{T}\text{A}{\%})$$

### Growth and nutrient utilization

Fish weight was measured at the beginning and end of the experiment using an electronic weighing balance (UniTech, Kolkata, India) to assess growth parameters. Before weighing, the fish were fasted 24 h. Feed and protein intake values were employed to calculate feed and nutrient utilization. The growth and nutrient utilization were calculated using the following formula^100,101^.

$$\:\text{W}\text{G}\left(\text{g}\right)=\text{F}\text{i}\text{n}\text{a}\text{l}\:\text{w}\text{e}\text{t}\:\text{w}\text{e}\text{i}\text{g}\text{h}\text{t}\:\left(\text{g}\right)-\text{I}\text{n}\text{i}\text{t}\text{i}\text{a}\text{l}\:\text{w}\text{e}\text{t}\:\text{w}\text{e}\text{i}\text{g}\text{h}\text{t}\left(\text{g}\right)$$

$$\:\text{W}\text{G}{\%}=\frac{\text{F}\text{i}\text{n}\text{a}\text{l}\:\text{w}\text{e}\text{t}\:\text{w}\text{e}\text{i}\text{g}\text{h}\text{t}\:\left(\text{g}\right)-\text{I}\text{n}\text{i}\text{t}\text{i}\text{a}\text{l}\:\text{w}\text{e}\text{t}\:\text{w}\text{e}\text{i}\text{g}\text{h}\text{t}\left(\text{g}\right)}{\text{I}\text{n}\text{i}\text{t}\text{i}\text{a}\text{l}\:\text{w}\text{e}\text{t}\:\text{w}\text{e}\text{i}\text{g}\text{h}\text{t}\left(\text{g}\right))}\text{x}\:\:10$$

$$\:\text{S}\text{G}\text{R}{\%}=\frac{\text{ln}\text{o}\text{f}\text{f}\text{i}\text{n}\text{a}\text{l}\:\text{w}\text{e}\text{t}\:\text{w}\text{e}\text{i}\text{g}\text{h}\text{t}\:\left(\text{g}\right)-\text{ln}\text{o}\text{f}\text{i}\text{n}\text{i}\text{t}\text{i}\text{a}\text{l}\:\text{w}\text{e}\text{t}\:\text{w}\text{e}\text{i}\text{g}\text{h}\text{t}\left(\text{g}\right)\:}{\text{E}\text{x}\text{p}\text{e}\text{r}\text{i}\text{m}\text{e}\text{n}\text{t}\text{a}\text{l}\:\text{p}\text{e}\text{r}\text{i}\text{o}\text{d}\:\left(\text{d}\text{a}\text{y}\text{s}\right)}\text{x}\:\:100$$

$$\:\text{F}\text{C}\text{R}=\frac{\text{F}\text{e}\text{e}\text{d}\:\text{i}\text{n}\text{t}\text{a}\text{k}\text{e}\:\left(\text{d}\text{r}\text{y}\:\text{w}\text{e}\text{i}\text{g}\text{h}\text{t}\:\text{i}\text{n}\:\text{g}\right)}{\text{B}\text{o}\text{d}\text{y}\text{w}\text{e}\text{i}\text{g}\text{h}\text{t}\:\text{g}\text{a}\text{i}\text{n}\:\left(\text{w}\text{e}\text{t}\:\text{w}\text{e}\text{i}\text{g}\text{h}\text{t}\:\text{i}\text{n}\:\text{g}\right)}\text{x}\:\:100$$

$$\:\text{P}\text{E}\text{R}=\frac{\text{B}\text{o}\text{d}\text{y}\text{w}\text{e}\text{i}\text{g}\text{h}\text{t}\:\text{g}\text{a}\text{i}\text{n}\:\left(\text{w}\text{e}\text{t}\:\text{w}\text{e}\text{i}\text{g}\text{h}\text{t}\:\text{i}\text{n}\:\text{g}\right)}{\text{P}\text{r}\text{o}\text{t}\text{e}\text{i}\text{n}\:\text{i}\text{n}\text{t}\text{a}\text{k}\text{e}\:\left(\text{d}\text{r}\text{y}\:\text{w}\text{e}\text{i}\text{g}\text{h}\text{t}\:\text{i}\text{n}\:\text{g}\right)}\text{x}\:\:100$$

### Digestibility study

A 30-day digestibility study, following a feeding trial, using chromic oxide (Cr₂O₃) as an inert marker. Fish were acclimated to test diets containing 0.5% Cr₂O₃ for a ten-day period prior to the trial. Daily feedings were administered at 10:00 AM, with subsequent fecal collection at 8:00 AM using a modified method. Collected feces were freeze-dried and stored at -20 °C for subsequent analysis. The samples’ crude protein, lipid, and ash content were determined^99^. The chromic oxide content in feed and fecal matter was measured^102^. Apparent digestibility coefficients (ADCs) for dry matter, protein, lipid, and carbohydrate were calculated^103^.

$$\:\text{A}\text{D}\text{C}\:\text{o}\text{f}\:\text{d}\text{r}\text{y}\:\text{m}\text{a}\text{t}\text{t}\text{e}\text{r}\:{\%}\:=\:100\:\times\:\left(1-\frac{{\%}\text{C}\text{h}\text{r}\text{o}\text{m}\text{i}\text{c}\:\text{o}\text{x}\text{i}\text{d}\text{e}\:\text{i}\text{n}\:\text{d}\text{i}\text{e}\text{t}\:\:}{{\%}\text{C}\text{h}\text{r}\text{o}\text{m}\text{i}\text{c}\:\text{o}\text{x}\text{i}\text{d}\text{e}\:\text{i}\text{n}\:\text{f}\text{e}\text{c}\text{e}\text{s}}\right)$$

$$\:\text{A}\text{D}\text{C}\:\text{o}\text{f}\:\text{n}\text{u}\text{t}\text{r}\text{i}\text{e}\text{n}\text{t}\text{s}\:{\%}\:=\:100\:\times\:\left(1-\frac{{\%}\text{C}\text{h}\text{r}\text{o}\text{m}\text{i}\text{c}\:\text{o}\text{x}\text{i}\text{d}\text{e}\:\text{i}\text{n}\:\text{d}\text{i}\text{e}\text{t}\:\text{x}\:{\%}\:\text{n}\text{u}\text{t}\text{r}\text{i}\text{e}\text{n}\text{t}\:\text{i}\text{n}\:\text{f}\text{e}\text{c}\text{e}\text{s}\:}{{\%}\text{C}\text{h}\text{r}\text{o}\text{m}\text{i}\text{c}\:\text{o}\text{x}\text{i}\text{d}\text{e}\:\text{i}\text{n}\:\text{f}\text{e}\text{c}\text{e}\text{s}\:\text{x}\:{\%}\:\text{n}\text{u}\text{t}\text{r}\text{i}\text{e}\text{n}\text{t}\:\text{i}\text{n}\:\text{t}\text{h}\text{e}\:\text{d}\text{i}\text{e}\text{t}}\right)$$

### Enzyme assays

#### Sample collection and tissue homogenate preparation

After the experiment, nine fish per group were anesthetized with clove oil (50 µl/L) and dissected to collect liver, intestine, and muscle tissues. Under cold conditions, these tissues were homogenized (5% in chilled 0.25 M sucrose) using a Teflon-coated mechanical homogenizer (HM30, REMI, India). The homogenate was then centrifuged (5000 rpm, 10 min, 4 °C) to separate the liquid portion (supernatant), which was stored frozen (-20 °C) for enzyme analysis.

#### Estimation of tissue protein

Lowry’s method measured Protein content in tissues^104^. Tissue samples (0.1 ml) were treated with an acid solution (TCA) and centrifuged to isolate the protein fraction. The remaining protein was then dissolved in a sodium hydroxide solution. This solution was mixed with specific chemicals and incubated in stages. The resulting solution’s colour intensity (optical density) was measured at 660 nm to determine protein concentration. A standard protein (BSA) helped calculate the actual protein amount in the fish tissues.

### Digestive enzyme activities

#### Protease

In 1974, a method was described for casein digestion to assess protein activity in intestine tissue homogenates^105^.

#### Amylase

The amylase activity of intestine tissue homogenate was determined using the DNS (3,5-dinitro salicylic acid) method^106^.

#### Lipase

Intestinal lipase activity was assessed using the titrimetric method^107^.

### Metabolic enzyme activities

Aspartate aminotransferase (AST) activities in the liver and muscle of *C. catla*were assayed^108^. The procedure for estimating alanine aminotransferase (ALT) activity mirrored that of AST activity, with the distinction that the substrate used was 0.2 M DL-alanine instead of L-aspartic acid. ALT activity was quantified as nanomoles of pyruvate produced per milligram of protein per minute at 37 °C. Lactate dehydrogenase (LDH) and Malate dehydrogenase (MDH) activity in various tissues was assessed^109,110^.

### Oxidative stress response

Superoxide dismutase (SOD) activity in liver tissue homogenates was quantified^111^, which is based on the enzyme’s inhibition of epinephrine oxidation to adrenochrome. Catalase (CAT) activity in liver tissue homogenates was determined^112^.

### Haematological and serum parameters

#### Blood and serum collection

Fish were anesthetized with clove oil (50 µL/L) prior to blood collection. Blood was drawn from the caudal vein and immediately transferred to EDTA coated vials to prevent clotting. Aliquots were used for hemoglobin (Hb), red blood cell (RBC), white blood cell (WBC), and nitroblue tetrazolium (NBT) assays. A separate blood sample was collected without anticoagulant, allowed to clot, and centrifuged. The resulting serum was stored at -20 °C for further biochemical and immunological analyses.

#### Serum glucose

Serum glucose levels were quantified using the Erba diagnostic kit (Transasia Bio-medicals Pvt. Ltd., India). The glucose oxidase oxidizes glucose to generate hydrogen peroxide (H_2_O_2_). The H_2_O_2_ subsequently reacts with 3,5-dichloro-2-hydroxybenzene sulfonic acid and 4-aminoantipyrine, forming a pink dye, and absorbance was measured at 514 nm.

#### Serum total protein

Blood serum protein was measured using a Biuret kit (Erba, India). The assay is based on the reaction of proteins with copper ions, resulting in a blue-violet complex whose intensity increases with protein concentration. The protein levels were quantified by measuring the absorbance of the solution at 546 nm.

#### Serum albumin

Serum albumin levels were quantified using the Erba diagnostic kit (Transasia Bio-medicals Pvt. Ltd., India). This method is based on the interaction between albumin and Bromocresol Green (BCG) (pH 4.2), which leads to a shift in the absorbance of the BCG dye. The resultant blue-green hue, directly proportional to the albumin concentration, was assessed within the 580–630 nm wavelength range.

#### Serum globulin

Serum globulin levels were determined by subtracting albumin levels from total protein content.

#### Albumin-globulin (A/G) ratio

The albumin to globulin ratio (A/G ratio) was calculated by dividing albumin levels by globulin levels.

#### Nitro blue tetrazolium (NBT) assay

Blood samples were drawn from the caudal vein into a test tube containing 2.7% EDTA. 50 µl of blood were then aliquoted into U-bottom microplate wells and incubated at 37 °C for 1 h to facilitate cell adhesion. Following incubation, the wells were rinsed with PBS and treated with 50 µl of 0.2% NBT for 1 h. Subsequently, the cells were fixed using 100% methanol for 2–3 min, rinsed with 30% methanol, and air-dried. A solution comprising 60 µl of 2 N KOH and 70 µl of dimethyl sulfoxide was added to each well to solubilize the formazan blue precipitate. The optical density (OD) was then measured at 450 nm using an ELISA reader^113^.

#### Haematological parameters

Blood parameters such as erythrocyte count (RBCs), hematocrit (PCV), hemoglobin (Hb), and leukocyte count (WBCs) were assessed using an Olympus BH-2 light microscope at 100× magnification^114^.

### Statistical analysis

The data were statistically analyzed using SPSS version 22.0. Normality was assessed via the Shapiro-Wilk test, and homoscedasticity was evaluated using Levene’s test. A one-way ANOVA was performed, followed by Duncan’s multiple-range test to determine significant differences among the means, with a significance level set at 5%.

## Conclusion

In summary, incorporating Anabaena blue-green algae (ABGA) into the diet of *Catla catla* fingerlings at levels up to 15% does not negatively impact growth performance. However, higher inclusion rates may induce adverse physiological and metabolic effects. Specifically, increased ABGA levels were associated with significant reductions in amylase activity, alterations in hematological parameters, elevated oxidative stress markers, and increased serum glucose levels. Thus, while up to 15% ABGA can be safely included in the diet without impairing growth, the physiological stress at higher concentrations requires further investigation to establish a safe and optimal inclusion level.

This study adhered to the guidelines set forth by the Committee for the Purpose of Control and Supervision of Experiments on Animals (CPCSEA), under the Ministry of Environment and Forests (Animal Welfare Division), Government of India, for the ethical care and use of animals in scientific research. All procedures involving fish handling and treatment received approval from the Ethics and Animal Care Committee of ICAR-CIFE, Mumbai, in compliance with institutional and national standards.

## Data Availability

The datasets used and/or analysed during the current study available from the corresponding author on reasonable request.
